# Draft Genome Sequence of *Halolamina pelagica* CDK2 Isolated from Natural Salterns from Rann of Kutch, Gujarat, India

**DOI:** 10.1128/genomeA.01593-16

**Published:** 2017-02-09

**Authors:** Sonam Gaba, Ram Nageena Singh, Shrutica Abrol, Ajar Nath Yadav, Anil Kumar Saxena, Rajeev Kaushik

**Affiliations:** Division of Microbiology, ICAR-Indian Agricultural Research Institute, Pusa, New Delhi, India

## Abstract

*Halolamina pelagica* strain CDK2, a halophilic archaeon (growth range 1.36 to 5.12 M NaCl), was isolated from rhizosphere of wild grasses of hypersaline soil of the Rann of Kutch, Gujarat, India. Its draft genome contains 2,972,542 bp and 3,485 coding sequences, depicting genes for halophilic serine proteases and trehalose synthesis.

## GENOME ANNOUNCEMENT

*Archaea* usually thrive in environments of extremity such as high temperature, salinity, or pH (1). The domain of *Archaea* is divided into six phyla (2–8). *Euryarchaeota* (4) is the most diverse of all the phyla occupying many different niches and includes the extreme halophiles (9). Halophilic archaea dwell in niches with high salt concentrations (1.5 M to 5 M NaCl) and have the ability to adjust intracellular solutes to adapt themselves to a hypersaline environment (10). We isolated *Halolamina pelagica* (11) strain CDK2 (12), a phosphorous solubilizing halophilic archaeon of the family *Haloferacaceae* (13), from Salterns of the Rann of Kutch, Gujarat, India. The genome sequence of *Halolamina pelagica* strain CDK2 (12) may lead to identification of genes imparting tolerance to salinity stress, which could be further utilized for various biotechnological applications following characterization.

Genomic DNA was extracted and purified using a DNA isolation kit from Zymo Research Laboratories and was sequenced using both the Miseq platform (Illumina) and PacBio SMRT version C5 chemistry. Shotgun sequencing generated 11,512,861 paired-end reads (2 × 250 bp), while PacBio SMRT resulted in 120,716 long reads (30 to 40 kb). The genome was assembled by the Celera Assembler program (14) using two approaches, hybrid (Illumina and PacBio reads) and nonhybrid assembly (PacBio reads). The hybrid approach resulted in 32 scaffolds, whereas nonhybrid assembly resulted in three scaffolds. The final draft genome was of 2,972,542 bp with G+C content of 67.6%, three scaffolds, and 3,550 predicted genes (including 3,485 coding sequences [CDSs]).

Many important annotated coding genes which could impart tolerance to high salinity, such as trehalose/maltose transport system permease (*malF* and *malG*), trehalose/maltose import ATP-binding protein (*malK*) and trehalose synthase, were found. Important antiporters involved in transport of sodium and hydrogen ions were found, like putative monovalent cation/H^+^ antiporter subunit C, D, E, and G, Na^+^/H^+^antiporter NhaC, and antiporter inner membrane protein, which play important roles in maintaining pH and sodium balance (15). Halophilic serine proteases such as halolysine precursor (15, 16) were also found. Genes responsible for phosphorus transport and regulation, such as phosphorus transporter permease subunit *pstA* and *pstC*, phosphate regulon (*pho*), phosphate regulon sensor kinase (*phoR*), and transcriptional regulator (*phoU*), were present in the genome. The genes for temperature tolerance include two genes (*cspE* and *cspC*) of cold-shock proteins and three genes (*cct2*, *cct1* and *htpX*) of heat-shock proteins (17, 18). CspE and CspC proteins are involved in stabilizing the transcript of the *rpoS* gene, coding for an important general stress response factor (19). Transcription induction studies of *cct1* and *cct*2 genes in archaea showed up- and downregulation of expression of transcripts with temperature (19).

Also noteworthy are the genes for copper transport such as putative copper-exporting P-type ATPase B and ATPase A, which were found to be involved in multi-copper enzyme maturation, maintaining copper homeostasis, and avoiding copper toxicity (20), suggesting that *Halolamina pelagica* strain CDK2 might be having a defined copper transport system. In addition to the above mentioned important genomic features, many archaeal specific signal peptides were also detected.

### Accession number(s).

This whole-genome project has been deposited at DDBJ/EMBL/GenBank under the accession number SUBID SUB1035521, BioProjectID PRJNA272888, BioSampleID SAMN03287580, and accession number LGUC00000000. This paper describes the first version.
